# Cochlear Implantation Outcomes in Genotyped Subjects with Sensorineural Hearing Loss

**DOI:** 10.1007/s10162-025-00987-0

**Published:** 2025-04-23

**Authors:** M. L. A. Fehrmann, L. Haer-Wigman, H. Kremer, H. G. Yntema, M. E. G. Thijssen, E. A. M. Mylanus, W. J. Huinck, C. P. Lanting, R. J. E. Pennings

**Affiliations:** 1 Department of Otorhinolaryngology, Radboud University Medical Center, Geert Grooteplein Zuid 10, 6525 GA Nijmegen, The Netherlands; 2 Donders Institute for Brain, Cognition and Behaviour, Nijmegen, The Netherlands; 3 Department of Human Genetics, Radboud University Medical Center, Nijmegen, The Netherlands

**Keywords:** Hereditary hearing loss, Genetics, Cochlear implantation outcomes, Clinical decision-making, Disease management

## Abstract

**Purpose:**

Cochlear implants (CIs) are an effective rehabilitation option for individuals with severe-to-profound sensorineural hearing loss (SNHL). While genetic factors play a significant role in SNHL, the variability in CI outcomes remains unclear. This study evaluated short- and long-term CI outcomes in a large genotyped cohort and investigated correlations with genetic defects and their cochlear site-of-lesion.

**Methods:**

This retrospective, single-center, cohort study included 220 subjects (127 females; 299 ears) with pathogenic variants identified in 31 different nuclear genes and in mitochondrial genes. Audiological outcomes were measured pre- and post-implantation. Cochlear site-of-lesion was categorized as pre-synaptic, post-synaptic, or mitochondrial, based on gene function or expression. Multiple regression analysis assessed factors influencing outcomes, including age at implantation, SNHL duration, hearing aid (HA) use, and cochlear site-of-lesion.

**Results:**

Results showed a median phoneme score of 90%, with better outcomes in early implantation (≤ 6 years). Variability in outcomes was not linked to cochlear site-of-lesion, but to subject-specific factors, such as age at implantation, duration of SNHL, pre-implantation HA use, and CI experience. A model incorporating these subject-specific factors explained 19% of the total variance in outcomes. Poorer outcomes (phoneme scores < 70%) were more common in individuals with prolonged auditory deprivation or older age at implantation.

**Conclusion:**

Genotyped CI recipients demonstrated excellent outcomes, with variability largely attributed to non-genetic factors. These findings show that cochlear implantation is a beneficial type of rehabilitation for most individuals with hereditary SNHL and underscore the importance of early implantation.

**Supplementary Information:**

The online version contains supplementary material available at 10.1007/s10162-025-00987-0.

## Introduction

Cochlear implantation is a successful type of rehabilitation for individuals with severe to profound sensorineural hearing loss (SNHL), enhancing hearing, speech recognition, and quality of life [1]. Cochlear implant (CI) performance is influenced by factors like cochlear anatomy, age at implantation, duration of SNHL, residual hearing, and cognitive performance [2–5]. Lazard et al. identified nine factors accounting for 22% of the variance in CI outcomes [4]. The top four factors, accounting for 10% of the variance, include duration of severe/profound SNHL, age of onset of severe/profound HL, duration of CI experience, and etiology [3]. This leaves 78% of the variance unexplained, with other contributing factors yet to be identified.

Genetic factors significantly contribute to SNHL, with 50–70% of cases with early-onset SNHL having a genetic cause [6]. Genetic SNHL is highly heterogeneous, involving hundreds of genes encoding proteins with roles in various parts within the auditory pathway and beyond. It can be syndromic (~ 30% of cases) or non-syndromic (~ 70% of cases) [7]. Syndromic SNHL is linked to other conditions, like retinitis pigmentosa (i.e., Usher syndrome), goiter (i.e., Pendred syndrome), or pigmentary anomalies of the skin, hair and eyes (i.e., Waardenburg syndrome) [8]. In contrast, non-syndromic SNHL occurs without additional symptoms. Currently, about 150 genes are linked to non-syndromic SNHL, with many more associated with syndromic SNHL [9]. Causative variants are commonly found in subjects with congenital or early-onset SNHL, but this decreases significantly with later onset of SNHL [10].

Several studies have examined the genetic etiology in CI recipients [11–17], with causative variants most frequently reported in *GJB2*, *SLC26 A4*, *TMPRSS3*, and *MYO7 A* (Supplementary Table 1). These four genes are associated with congenital severe-to-profound SNHL, although *TMPRSS3* is also linked to post-lingual onset high-frequency SNHL [18, 19]. While *GJB2* and *TMPRSS3* are associated with non-syndromic SNHL, *MYO7 A* and *SLC26 A4* are linked to both non-syndromic (DFNA11/DFNB2 and DFNB4) and syndromic SNHL (Usher syndrome type 1b and Pendred syndrome).

Studies evaluating CI outcomes in genotyped recipients generally report favorable outcomes [13, 16, 19–21]. Yet, two studies noted significant variation in success rates based on genotype [16, 21]. Tropitzsch et al. observed that subjects with variants affecting the neural component of the cochlea had CI outcomes 35% worse than the overall median score [16], supporting the spiral ganglion hypothesis proposed by Eppsteiner et al. According to this hypothesis, variability in CI outcomes is linked to the health of the spiral ganglion neurons (SGNs) and cochlear nerve [22]. SGNs transmit information from inner hair cells (IHCs) and outer hair cells (OHCs) [23–25] through their axons, which form the cochlear nerve [26, 27], to the cochlear nucleus [23, 24]. Because a CI directly stimulates the SGNs, poorer CI outcomes are anticipated when these neural components of the auditory pathway are affected. Conversely, good performance is anticipated when pre-synaptic structures, such as the organ of Corti and/or the stria vascularis, are affected [22].

This study aimed to evaluate CI outcomes in fully genotyped SNHL subjects within a large Dutch cohort. The primary objective was to assess CI outcomes at short- and long-term intervals. Additionally, we aimed to investigate whether the causative genetic defects contribute to the variability in CI outcomes by comparing outcomes between genes and cochlear site-of-lesions.

## Methods and Materials

### Study Design and Population

This retrospective, observational cohort study evaluated CI performance in subjects with hereditary SNHL. Ethical approval for this study was granted by the Medical Ethics Committee (METC) Eastern Netherlands. The requirement for signed informed consent was waived by the ethics committee because all data were collected, saved, analyzed, and reported anonymously. All genotyped individuals who underwent cochlear implantation at our center from January 2002 to March 2021 were identified. Subsequently, subjects were included in the study when they (1) had a confirmed genetic diagnosis based on monoallelic or biallelic (likely) pathogenic variants in, respectively, dominantly or recessively, inherited genes associated with SNHL; (2) underwent cochlear implantation after 2002; (3) had at least 1 year of follow-up for speech recognition measurements post-implantation. Individuals with syndromic SNHL and developmental delays that could potentially interfere with CI performance (e.g., CHARGE syndrome, Charcot-Marie-Tooth disease, and Noonan syndrome) were excluded. Some subjects enrolled in this study were described in previous publications [28–34].

### Data Collection

Demographic data were obtained by reviewing medical records, including sex, use of hearing aids (HA) prior to implantation, age at implantation, and type of implant and electrode used. Pre-implantation imaging was reviewed to assess inner ear anatomy, and the type of insertion was scored to evaluate surgical factors.

#### Genotype and Cochlear Site-of-Lesion

No new genetic analyses were performed for this study. Genotype data were obtained from previous genetic tests performed in the past two decades in a clinical setting. Subjects were seen in our outpatient clinic for genetic diagnostic screening and/or cochlear implantation. The type of genetic test varied per case, depending on the phenotype and family history, and included both targeted gene sequencing (< 2015) and/or whole-exome sequencing (WES, > 2014). The identified variant(s) in genes associated with SNHL, along with the corresponding protein change(s) and type of variant (truncating or missense), were documented from the original DNA-diagnostic laboratory reports. Given that variant classifications may have changed over time, all identified variants were re-classified according to the current ACMG-AMP variant classification guidelines by an experienced molecular lab specialist to ensure the most up-to-date categorization [35]. Subjects without monoallelic or biallelic (likely) pathogenic variants under these guidelines in dominantly or recessively inherited genes were excluded upon reclassification.

The cochlear site-of-lesion for the genes was determined by evaluating the encoded protein function as reported in current literature. If this was unknown, we considered the currently reported expression patterns (Table 1). Genes were classified into pre-synaptic (e.g., hair cells (HC), stria vascularis or tectorial membrane), post-synaptic (SGN or cochlear nerve), and mitochondrial groups. The mitochondrial group included subjects with pathogenic variants in mitochondrial DNA (mtDNA) or genes mainly affecting mitochondrial function. Genes associated with both pre- and post-synaptic pathology were excluded from the cochlear site-of-lesion classification.

**Table 1:** Pathogenic variants in the study population

Gene	Transcript	cDNA	Protein	Type of mutation	Classification	Cochlear site-of-lesion	Group	References^**^
*ACTB*	NM_001101.3	c.547 C > T	p.(Arg183 Trp)	Missense	Pathogenic	Hair cell	Pre-synaptic	[36, 37]
*ACTG1*	NM_001614.3	c.151G > A	p.(Asp51 Asn)	Missense	Pathogenic	Hair cell	Pre-synaptic	[38]
*ACTG1*	NM_001614.5	c.833 C > T	p.(Thr278Ile)	Missense	Pathogenic	Hair cell	Pre-synaptic	
*ADGRV1*	NM_032119.4	c.8875 C > T	p.(Arg2959*)	Truncating	Pathogenic	Hair cell	Pre-synaptic	[39]
*CDH23*	NM_022124.6	c.3706 C > T	p.(Arg1236*)	Truncating	Pathogenic	Hair cell	Pre-synaptic	[40, 41]
*CDH23*	NM_022124.6	c.3955G > T	p.(Glu1319*)	Truncating	Pathogenic	Hair cell	Pre-synaptic	
*CDH23*	NM_022124.6	c.6050 - 9G > A	p.(Val2018fs)	Truncating	Pathogenic	Hair cell	Pre-synaptic	
*CLRN1*	NM_174878.3	c.149_152 delinsTGTCCAAT	p.(Ser50fs)	Truncating	Pathogenic	*	*	
*CLRN1*	NM_174878.3	c.528 T > G	p.(Tyr176*)	Truncating	Pathogenic	*	*	
*COCH*	NM_004086.2	c.151 C > T	p.(Pro51Ser)	Missense	Pathogenic	*	Pre-synaptic	[42]
*COCH*	NM_004086.2	c.263G > A	p.(Gly88Glu)	Missense	Pathogenic	*	Pre-synaptic	
*EDNRB*	NM_000115.5	c.1195 - 1G > A	r.spl	Missense	Pathogenic	Stria vascularis	Pre-synaptic	[43]
*GJB2*	NM_004004.6	c.− 23 + 1G > A	p.0	Missense	Pathogenic	Ion homeostasis	Pre-synaptic	[44]
*GJB2*	NM_004004.6	c.139G > T	p.(Glu47*)	Truncating	Pathogenic	Ion homeostasis	Pre-synaptic	
*GJB2*	NM_004004.5	c.223 C > T	p.(Arg75 Trp)	Missense	Pathogenic	Ion homeostasis	Pre-synaptic	
*GJB2*	NM_004004.6	c.229 T > C	p.(Trp77 Arg)	Missense	Pathogenic	Ion homeostasis	Pre-synaptic	
*GJB2*	NM_004004.5	c.235 del	p.(Leu79fs)	Truncating	Pathogenic	Ion homeostasis	Pre-synaptic	
*GJB2*	NM_004004.6	c.238 C > A	p.(Gln80Lys)	Missense	Pathogenic	Ion homeostasis	Pre-synaptic	
*GJB2*	NM_004004.6	c.35 del	p.(Gly12fs)	Truncating	Pathogenic	Ion homeostasis	Pre-synaptic	
*GJB2*	NM_004004.6	c.427 C > T	p.(Arg143 Trp)	Missense	Pathogenic	Ion homeostasis	Pre-synaptic	
*GJB2*	NM_004004.5	c.71G > A	p.(Trp24*)	Missense	Pathogenic	Ion homeostasis	Pre-synaptic	
*GJB2*	NM_004004.6	c.313_326 del	p.(Lys105fs)	Truncating	Pathogenic	Ion homeostasis	Pre-synaptic	
*GJB2*	NM_004004.6	c.32_45 del	p.(Gly11fs)	Truncating	Pathogenic	Ion homeostasis	Pre-synaptic	
*GJB2*	NM_004004.5	c.407 dup	p.(Tyr136*)	Truncating	Pathogenic	Ion homeostasis	Pre-synaptic	
*GJB2*	NM_004004.5	c.551G > A	p.(Arg184Gln)	Missense	Pathogenic	Ion homeostasis	Pre-synaptic	
*GJB6*		c.del(GJB6-D13S1830)	del(GJB6-D13S1830)	Truncating	Pathogenic	Ion homeostasis	Pre-synaptic	
*LARS2*	NM_015340.3	c.683G > A	p.(Arg228His)	Missense	Pathogenic	Mitochondrial	Mitochondrial	[45]
*LARS2*	NM_015340.3	c.880G > A	p.Glu294Lys	Missense	Pathogenic	Mitochondrial	Mitochondrial	
*MITF*	NM_000248.3	c.649 del	p.(Arg217fs)	Truncating	Pathogenic	Stria vascularis	Pre-synaptic	[46]
*MITF*	NM_000248.3	c.772 C > T	p.(Gln258*)	Truncating	Pathogenic	Stria vascularis	Pre-synaptic	
*Mitochondrial*		m.03243 A > G			Pathogenic	Mitochondrial	Mitochondrial	[47, 48]
*Mitochondrial*		m.07472 insC			Pathogenic	Mitochondrial	Mitochondrial	
*MYH9*	NM_002473.4	c.2507 C > T	p.(Pro836Leu)	Missense	Likely pathogenic	Hair cell	Pre-synaptic	[49]
*MYO15 A*	NM_016239.3	c.10082 + 1G > A	r.spl	Missense	Pathogenic	Hair cell	Pre-synaptic	[50]
*MYO15 A*	NM_016239.3	c.10216 + 1G > A	r.spl	Missense	Pathogenic	Hair cell	Pre-synaptic	
*MYO15 A*	NM_016239.3	c.10258_10260 del	p.(Phe3420 del)	Truncating	Pathogenic	Hair cell	Pre-synaptic	
*MYO15 A*	NM_016239.3	c.1137 del	p.(Tyr380fs)	Truncating	Pathogenic	Hair cell	Pre-synaptic	
*MYO15 A*	NM_016239.3	c.3742 C > T	p.(Arg1248 Trp)	Missense	Likely pathogenic	Hair cell	Pre-synaptic	
*MYO15 A*	NM_016239.3	c.4519 C > T	p.(Arg1507*)	Truncating	Pathogenic	Hair cell	Pre-synaptic	
*MYO15 A*	NM_016239.3	c.6787G > A	p.(Gly2263Ser)	Missense	Likely pathogenic	Hair cell	Pre-synaptic	
*MYO15 A*	NM_016239.3	c.6892 C > T	p.(Arg2298*)	Truncating	Pathogenic	Hair cell	Pre-synaptic	
*MYO15 A*	NM_016239.3	c.7893 + 1G > A	r.spl	Missense	Pathogenic	Hair cell	Pre-synaptic	
*MYO15 A*	NM_016239.3	c.806 dup	p.(Ala270fs)	Truncating	Pathogenic	Hair cell	Pre-synaptic	
*MYO15 A*	NM_016239.3	c.2471 del	p.(Pro824fs)	Truncating	Pathogenic	Hair cell	Pre-synaptic	
*MYO15 A*	NM_016239.3	c.625G > T	p.(Glu209*)	Truncating	Pathogenic	Hair cell	Pre-synaptic	
*MYO15 A*	NM_016239.3	c.8968 - 1G > T	r.spl	Missense	Pathogenic	Hair cell	Pre-synaptic	
*MYO3 A*	NM_017433.4	c.2090 T > G	p.(Leu697 Trp)	Missense	Pathogenic	Hair cell	Pre-synaptic	[51]
*MYO7 A*	NM_000260.4	c.1522 T > C	p.(Ser508Pro)	Missense	Likely pathogenic	Hair cell	Pre-synaptic	[52]
*MYO7 A*	NM_000260.4	c.2078 del	p.(Lys693fs)	Truncating	Likely pathogenic	Hair cell	Pre-synaptic	
*MYO7 A*	NM_000260.4	c.3039 dup	p.(Thr1014fs)	Truncating	Likely pathogenic	Hair cell	Pre-synaptic	
*MYO7 A*	NM_000260.4	c.3109 - 2 A > G	r.spl	Splice	Pathogenic	Hair cell	Pre-synaptic	
*MYO7 A*	NM_000260.4	c.3508G > A	p.(Glu1170Lys)	Missense	Pathogenic	Hair cell	Pre-synaptic	
*MYO7 A*	NM_000260.4	c.3719G > A	p.(Arg1240Gln)	Missense	Pathogenic	Hair cell	Pre-synaptic	
*MYO7 A*	NM_000260.4	c.3764 del	p.(Lys1255fs)	Truncating	Pathogenic	Hair cell	Pre-synaptic	
*MYO7 A*	NM_000260.4	c.4117 C > T	p.(Arg1373*)	Truncating	Pathogenic	Hair cell	Pre-synaptic	
*MYO7 A*	NM_000260.4	c.5648G > A	p.(Arg1883Gln)	Missense	Pathogenic	Hair cell	Pre-synaptic	
*MYO7 A*	NM_000260.4	c.5944G > A	r.5857_5944 del p.(Val1953fs)	Splice	Pathogenic	Hair cell	Pre-synaptic	
*MYO7 A*	NM_000260.4	c.6028G > A	p.(Asp2010 Asn)	Missense	Pathogenic	Hair cell	Pre-synaptic	
*MYO7 A*	NM_000260.4	c.1373 A > G	p.(Asn458Ser)	Missense	Likely pathogenic	Hair cell	Pre-synaptic	
*MYO7 A*	NM_000260.4	c.1373 A > T	p.(Asn458Ile)	Missense	Likely pathogenic	Hair cell	Pre-synaptic	
*MYO7 A*	NM_000260.4	c.5573 T > C	p.(Leu1858Pro)	Missense	Pathogenic	Hair cell	Pre-synaptic	
*OPA1*	NM_130837.2	c.1499G > A	p.(Arg500His)	Missense	Pathogenic	Auditory nerve	Post-synaptic	[53]
*OTOF*	NM_194248.2	c.2122 C > T	p.(Arg708*)	Truncating	Pathogenic	Hair cell	Pre-synaptic	[54]
*OTOF*	NM_194248.2	c.2649 C > A	p.(Cys883*	Truncating	Pathogenic	Hair cell	Pre-synaptic	
*OTOF*	NM_194248.2	c.3264_3274 dup	p.(Phe1092fs)	Truncating	Pathogenic	Hair cell	Pre-synaptic	
*PAX3*	NM_181457.3	c.1084_1087 dup	p.(Phe363fs)	Truncating	Pathogenic	Stria vascularis	Pre-synaptic	[55]
*PAX3*	NM_181457.3	c.242G > T	p.(Gly81 Val)	Missense	Likely pathogenic	Stria vascularis	Pre-synaptic	
*PAX3*	NM_181457.3	c.246_247 del	p.(Val83fs)	Truncating	Pathogenic	Stria vascularis	Pre-synaptic	
*PCDH15*	NM_001142769.2	c.4542 dup	p.(Pro1515fs)	Truncating	Likely pathogenic	Hair cell	Pre-synaptic	[56]
*POU4 F3*	NM_002700.3	c.668 T > C	p.(Leu223Pro)	Missense	Pathogenic	Hair cell	Pre-synaptic	[57]
*PTPRQ*	GW420685	c.1491 T > A	p.(Tyr497*)	Truncating	Pathogenic	Hair cell	Pre-synaptic	[58]
*PTPRQ*	GW420685	c.1548 del	p.(Tyr516*)	Truncating	Pathogenic	Hair cell	Pre-synaptic	
*SLC26 A4*	NM_000441.1	c.− 3- 25_164 + 160 del	p.?	Truncating	Pathogenic	Ion homeostasis	Pre-synaptic	[39, 59]
*SLC26 A4*	NM_000441.1	c.1001 + 1G > A	r.spl	Splice	Pathogenic	Ion homeostasis	Pre-synaptic	
*SLC26 A4*	NM_000441.1	c.1147 del	p.(Gln383fs)	Truncating	Pathogenic	Ion homeostasis	Pre-synaptic	
*SLC26 A4*	NM_000441.1	c.1172G > A	p.(Ser391 Asn)	Missense	Likely pathogenic	Ion homeostasis	Pre-synaptic	
*SLC26 A4*	NM_000441.1	c.1174 A > T	p.(Asn392 Tyr)	Missense	Pathogenic	Ion homeostasis	Pre-synaptic	
*SLC26 A4*	NM_000441.1	c.1246 A > C	p.(Thr416Pro)	Missense	Pathogenic	Ion homeostasis	Pre-synaptic	
*SLC26 A4*	NM_000441.1	c.1489G > A	p.(Gly497Ser)	Missense	Pathogenic	Ion homeostasis	Pre-synaptic	
*SLC26 A4*	NM_000441.1	c.2 T > C	p.(Met1?)	Missense	Pathogenic	Ion homeostasis	Pre-synaptic	
*SLC26 A4*	NM_000441.1	c.412G > T	p.(Val138Phe)	Missense	Pathogenic	Ion homeostasis	Pre-synaptic	
*SLC26 A4*	NM_000441.1	c.706 C > G	p.(Leu236 Val	Missense	Pathogenic	Ion homeostasis	Pre-synaptic	
*SLC26 A4*	NM_000441.1	c.707 C > T	p.(Leu236Pro)	Missense	Pathogenic	Ion homeostasis	Pre-synaptic	
*SLC26 A4*	NM_000441.1	c.754 T > C	p.(Ser252Pro)	Missense	Pathogenic	Ion homeostasis	Pre-synaptic	
*SLC26 A4*	NM_000441.1	c.1342 - 2_1343 dup	p.(Leu450fs)	Truncating	Pathogenic	Ion homeostasis	Pre-synaptic	
*SLC26 A4*	NM_000441.1	c.1694G > A	p.(Cys565 Tyr)	Missense	Pathogenic	Ion homeostasis	Pre-synaptic	
*SLC26 A4*	NM_000441.1	c.1991 C > T	p.(Ala664 Val)	Missense	Pathogenic	Ion homeostasis	Pre-synaptic	
*SLC26 A4*	NM_000441.1	c.2048 T > C	p.(Phe683Ser)	Missense	Pathogenic	Ion homeostasis	Pre-synaptic	
*SMPX*	NM_014332.1	c.45 + 1G > C	p.?	Splice	Pathogenic	Hair cell	Pre-synaptic	[60]
*SOX10*	NM_006941.3	c.472G > T	p.(Glu158*)	Truncating	Pathogenic	Ion homeostasis	Pre-synaptic	[61]
*SOX10*	NM_006941.3	c.1229_1235 delinsTGGCC	p.(Pro410LeufsTer31)	Truncating	Pathogenic	Ion homeostasis	Pre-synaptic	
*TMC1*	NM_138691.2	c.229 del	p.(Arg77fs)	Truncating	Pathogenic	Hair cell	Pre-synaptic	[62]
*TMC1*	NM_138691.2	c.1763 + 3 A > G	p.(Met589fs)	Truncating	Pathogenic	Hair cell	Pre-synaptic	
*TMC1*	NM_138691.2	c.65 - 1G > C	r.spl	Missense	Likely pathogenic	Hair cell	Pre-synaptic	
*TMPRSS3*	NM_024022.4	c.1276G > A	p.(Ala426 Thr)	Missense	Likely pathogenic	Hair cell	Presynaptic	(63)
*TMPRSS3*	NM_024022.4	c.208 del	p.(His70fs)	Truncating	Pathogenic	Hair cell	Presynaptic	
*TMPRSS3*	NM_024022.4	c.323 - 6G > A	p.(Val108Glyfs)	Truncating	Pathogenic	Hair cell	Presynaptic	
*TMPRSS3*	NM_024022.4	c.413 C > A	p.(Ala138Glu)	Missense	Likely pathogenic	Hair cell	Presynaptic	
*TMPRSS3*	NM_024022.4	c.595G > A	p.(Val199Met)	Missense	Likely pathogenic	Hair cell	Presynaptic	
*TMPRSS3*	NM_024022.4	c.916G > A	p.(Ala306 Thr)	Missense	Likely pathogenic	Hair cell	Presynaptic	
*TMPRSS3*	NM_024022.4	c.936 del	p.(Pro313fs)	Truncating	Likely pathogenic	Hair cell	Presynaptic	
*TMPRSS3*	NM_024022.4	c.325 C > T	p.(Arg109 Trp)	Missense	Pathogenic	Hair cell	Presynaptic	
*TPRN*	NM_001128228.2	c.1530 del	p.(Thr511fs)	Truncating	Pathogenic	Hair cell	Presynaptic	[64]
*TPRN*	NM_001128228.2	c.225_235 del	p.(Gly76fs)	Truncating	Pathogenic	Hair cell	Presynaptic	
*TPRN*	NM_001128228.2	c.744G > A	p.(Trp248*)	Truncating	Pathogenic	Hair cell	Presynaptic	
*TUBB4B*	NM_006088.6	c.729_736 delinsCGGCCAGA	p.(Leu246Ile)	Missense	Likely pathogenic	Hair cell	Presynaptic	[65]
*USH1 C*	NM_153676.4	c.364 C > T	p.(Gln122*)	Truncating	Pathogenic	Hair cell	Presynaptic	[66, 67]
*USH1 C*	NM_153676.3	c.497 - 2 del	r.spl	Truncating	Pathogenic	Hair cell	Presynaptic	
*USH2 A*	NM_206933.4	c.11864G > A	p.(Trp3955*)	Truncating	Pathogenic	Hair cell	Presynaptic	[67, 68]
*USH2 A*	NM_206933.4	c.1256G > T	p.(Cys419Phe)	Missense	Pathogenic	Hair cell	Presynaptic	
*USH2 A*	NM_206933.4	c.14289 del	p.(Ile4764fs)	Truncating	Pathogenic	Hair cell	Presynaptic	
*USH2 A*	NM_206933.4	c.1606 T > C	p.(Cys536 Arg)	Missense	Pathogenic	Hair cell	Presynaptic	
*USH2 A*	NM_206933.4	c.2299 del	p.(Glu767fs)	Truncating	Pathogenic	Hair cell	Presynaptic	
*USH2 A*	NM_206933.4	c.5018 T > C	p.(Leu1673Pro)	Missense	Pathogenic	Hair cell	Presynaptic	
*USH2 A*	NM_206933.4	c.7121 - 8313_11048 - 962 delins12	p.(Val2374_Gly3683 del)	Deletion	Pathogenic	Hair cell	Presynaptic	
*USH2 A*	NM_206933.4	c.8079G > A	p.(Trp2693*)	Truncating	Pathogenic	Hair cell	Presynaptic	
*USH2 A*	NM_206933.4	c.949 C > A	r.951_1143 del p.(Tyr318fs)	Missense	Pathogenic	Hair cell	Presynaptic	
*USH2 A*	NM_206933.4	c.10525 A > T	p.(Lys3509*)	Truncating	Pathogenic	Hair cell	Presynaptic	
*USH2 A*	NM_206933.4	c.6325 + 1G > C	r.spl	Splice	Pathogenic	Hair cell	Presynaptic	
*USH2 A*	NM_206933.4	c.8723_8724 del	p.(Val2908fs)	Truncating	Pathogenic	Hair cell	Presynaptic	
*WFS1*	NM_006005.3	c.2051 C > T	p.(Ala684 Val)	Missense	Pathogenic	*	*	[69]
*WFS1*	NM_006005.3	c.2115G > C	p.(Lys705 Asn)	Missense	Pathogenic	*	*	
*WFS1*	NM_006005.3	c.2508G > C	p.(Lys836 Asn)	Missense	Likely pathogenic	*	*	
*WFS1*	NM_006005.3	c.2590G > A	p.(Glu864Lys)	Missense	Pathogenic	*	*	

^*^Cochlear site-of-lesion could not be determined

^**^References for the cochlear site-of-lesion

#### Audiological Performance

Audiometry data were retrieved from medical records. Standard pure tone and speech audiometry were conducted per local protocols. The pure tone average (PTA) was determined using thresholds at 500, 1000, 2000, and 4000 Hz (PTA_0.5-4kHz_). Phoneme scores were assessed in quiet at 65 dB SPL. Both unaided and hearing-aid-assisted PTA and phoneme scores were measured pre-implantation. Post-implantation, unaided PTA scores, and CI-aided PTA and phoneme scores were measured. The post-implantation PTA_0.5-4kHz_ and phoneme scores at 65 dB SPL were evaluated at one year, five years, and long-term (≥ 6 years post-implantation).

Not all subjects used hearing aids (HA) prior to implantation. We calculated the best-aided PTA and phoneme score to represent the pre-implantation auditory performance. The best-aided scores were derived from the scores obtained while using a HA in the implanted ear or from unaided scores in those subjects not using a HA preoperatively. These scores were used to compare pre-implantation hearing performance with post-implantation CI Performance.

In early-implanted subjects with prelingual SNHL, behavioral observation audiometry (BOA) or visual reinforcement audiometry (VRA), was used for pre-implantation and one-year follow-up pure tone thresholds. Due to their young age, speech audiometry could not be performed pre-implantation or at one-year follow-up for these children.

### Data Analysis

Statistical analyses were performed with the IBM Statistical Package for the Social Science Statistics (SPSS) version 29, with a p-value < 0.05 considered statistically significant. Continuous variables were expressed as median with interquartile ranges (IQR) due to non-normal distribution as tested with the Shapiro–Wilk test. The Wilcoxon signed-rank test compared mean PTA and phoneme scores at different follow-up moments.

Because of the broad variability in the age of implantation, CI outcomes were separately evaluated for early-implanted (first CI ≤ 6 years of age) and late-implanted subjects (first CI ≥ 7 years of age). This cut-off was based on research indicating that the effects of auditory deprivation on the nervous system can be (partially) restored up to age seven [70, 71].

To determine which factors contribute to the variability in CI outcomes, we first performed univariate regression analysis including the following variables: gender, age at implantation, self-reported duration of SNHL, duration of CI experience, use of HA in the ear to be implanted, degree of SNHL pre-implantation, residual hearing (unaided PTA_0.5-4kHz_ measured two (IQR 1–7) months post-implantation), type of electrode array (perimodiolar (PME) versus lateral wall electrode (LWE)), implant brand (Cochlear LTD, Advanced Bionics, Oticon, MED-EL), year of implantation, type of insertion (cochleostomy, round window, extended round window), and cochlear site-of-lesion (HC, stria vascularis, ion homeostasis, cochlear nerve, mitochondria). Additionally, multiple regression analyses assessed the variance explained by each factor. A prediction model was constructed using backward selection, where factors within the model were deemed to significantly contribute with a *p*-value < 0.05. Sex- and gender-based analyses were conducted by incorporating gender as a contributing factor in these analyses.

The Mann–Whitney *U* test compared phoneme scores between two different subgroups. CI recipients with a measured phoneme score < 70% during the last follow-up visit were considered poorer performers and underwent more detailed evaluation, including a thorough review of their medical history, peri-implantation course, and rehabilitation period.

## Results

### Study Population

**Fig. 1:**
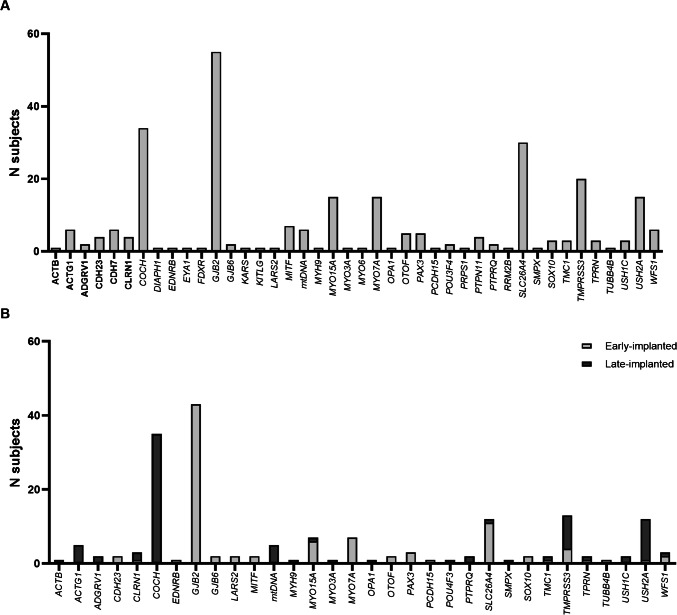
Distribution of affected genes. **A** Distribution of affected genes among the identified genotyped CI-recipients (*N* = 274 subjects). **B** Distribution of affected genes among the study population meeting the inclusion criteria (*N* = 220 subjects). Early-implanted denotes subjects who received their first CI at age ≤ 6 years, while late implanted indicates the first cochlear implantation performed at age > 6 years

Between 2002 and 2021, 274 genotyped individuals underwent cochlear implantation. Causal variants were identified in 41 different nuclear and mitochondrial genes, with *GJB2* (20.1%), *COCH* (12.5%), *SLC26 A4* (10.9%), and *TMPRSS3* (7.3%) being the most commonly affected genes (Fig. 1A). After evaluation of in- and exclusion criteria, 220 subjects were included in this study (Table 2). Nine subjects were excluded because of missing data, and 35 subjects because they, upon reclassification of identified variants, had no monoallelic or biallelic (likely) pathogenic variants in, respectively, dominantly or recessively inherited genes associated with SNHL. Additionally, ten subjects with syndromic SNHL linked to developmental delays that could potentially interfere with CI performance were excluded (CHARGE syndrome (*N* = 5); Charcot-Marie-Tooth disease (*N* = 1); Noonan syndrome (*N* = 4)).

**Table 2:** Subject characteristics

Subject characteristic	Total study cohort *N* = 220 subjects (100%)	
Gender, % female	127	(57.7)
Implantation		
Unilateral	141	(64.1)
Bilateral simultaneously	40	(18.2)
Bilateral sequentially	39	(17.7)
Ear characteristics	***N* = 299 ears (100%)**	
Age at implantation (median with IQR)	6 y (1–43)	
Self-reported duration of hearing loss prior to implantation (median with IQR)	5 y (1–20)	
Deviating anatomy at CT or MRI		
Normal anatomy	260	(87.0)
Enlarged vestibular aqueduct	34	(11.4)
Cochlear incomplete partition	4	(1.3)
Hypoplasia of the cochlear nerve	1	(0.3)
Degree HL pre-implantation*		
Moderate (41–60 dB HL)	4	(1.3)
Severe (61–80 dB HL)	23	(7.7)
Profound (> 80 dB HL)	237	(79.3)
Missing	35	(11.7)
Hearing aid in ear to be implanted	264	(88.3)
Surgical technique		
Cochleostomy	181	(60.5)
Round window	99	(33.1)
Extended round window	17	(5.7)
Not reported	2	(0.7)
Type of electrode		
PME	200	(66.9)
LWE	99	(33.1)

*IQR* interquartile range, *y* years, *CT* computer tomography, *MRI* magnetic resonance imaging, *HL* hearing loss, *PME* peri-modiolar electrode array, *LWE* lateral wall electrode array

^*^According to WHO’s grades of hearing impairment

Within the study population, (likely) pathogenic variants were identified in 31 different nuclear and mitochondrial genes (Fig. 1B). The most frequently affected genes were *GJB2* (23.6%), *COCH* (15.9%), and *SLC26 A4* (10.5%). Within the 220 subjects included, 124 unique variants were identified (Table 1), and 299 cochlear implantations were performed, with 141 subjects receiving unilateral implants and 79 receiving bilateral implants (40/79 simultaneous and 39/79 sequential).

### Cochlear Implantation Outcomes

Pre- and post-implantation PTA_0,5-4kHz_ and phoneme scores at 65 dB HL in quiet are shown in Table 3. The median last measured phoneme score was 90% (IQR 80–98). In late-implanted subjects (*N* = 143 ears), the last measured median phoneme score of 83% (IQR 74–90) was significantly lower than in early-implanted subjects (*N* = 156 ears) with a median score of 96% (IQR 90–100; *p* < 0.001). Early-implanted subjects also showed less variability in phoneme scores than the late-implanted subjects (Table 3).

**Table 3:** Cochlear implantation outcomes

		Total study population *N* = 299 ears		Early-implanted subjects *N* = 156 ears		Late-implanted subjects *N* = 143	
*Age at implantation*		6 y (1–43 y)		15 m (10–37 m)		44 y (17–60 y)	
*PTA*_*0,5-4kHz*_* (dB HL)*							
*Pre-implantation*	Unaided PTA_0,5-4kHz_	101 (90–111)	*N* = 264	106 (91–113)	*N* = 122	99 (86–110)	*N* = 142
	Best-aided PTA_0,5-4kHz_	73 (51–90)	*N* = 294	80 (70–95)	*N* = 152	59 (48–81)	*N* = 142
*Post-implantation*	Aided PTA_0,5-4kHz_ 1y FU	29 (25–34)*	*N* = 287	31 (27–38)*	*N* = 151	26 (24–30)*	*N* = 136
	Aided PTA_0,5-4kHz_ 5y FU	24 (21–26)*	*N* = 195	24 (21–26)*	*N* = 143	26 (24–30)	*N* = 52
	Aided PTA_0,5-4kHz_ LT FU	23 (21–25)^+^	*N* = 137	23 (20–25)^+^	*N* = 107	25 (23–32)	*N* = 30
*Phoneme scores (%)*							
*Pre-implantation*	Unaided phoneme score	0 (0–0)	*N* = 144	0 (0–10)	*N* = 11	0 (0–0)	*N* = 133
	Best-aided phoneme score	32 (0–58)	*N* = 144	50 (0–55)	*N* = 11	30 (0–59)	*N* = 133
*Post-implantation*	Aided phoneme score 1y FU	83 (72–90)*	*N* = 170	85 (72–95)^+^	*N* = 33	83 (72–90)*	*N* = 137
	Aided phoneme score 5y FU	93 (85–97)*	*N* = 217	95 (90–98)^+^	*N* = 144	87 (74–94)^+^	*N* = 73
	Aided phoneme score LT FU	95 (86–100)^+^	*N* = 146	96 (95–100)*	*N* = 107	85 (77–92)^+^	*N* = 39

*y* years, *m* months, *PTA* pure tone average, *FU* follow-up, *LT* long term

Ages, PTA_0,5-4kHz_, and phoneme scores are presented as median with inter quartile ranges (IQR). Long-term follow-up was 10.1 (IQR 8.3–13.4) years

^*^Significant improvement compared to previous measurement (*p* < 0.001)

^+^Significant improvement compared to previous measurement (*p* < 0.010)

### Cochlear Implantation Outcomes in Relation to the Cochlear Site-of-Lesion

Of the 31 affected nuclear genes, 28 were classified as either pre-synaptic or post-synaptic (Table 1). *CLRN1* (*N* = 6 ears) and *WFS1* (*N* = 7 ears) were excluded due to association with both pre- and post-synaptic pathology [72–74]. The phenotypes of these individuals have been described in detail in previous publications [33, 75]. Among them, only one individual with Usher syndrome type 3 exhibited bilateral hypoplasia of the cochlear nerve. The remaining subjects with *CLRN1*- or *WFS1*-associated SNHL showed no evidence of auditory neuropathy, as their CI outcomes remained stable over many years follow-up, as described in prior publications [33, 75].

Because of the widespread expression of *COCH* in the inner ear*,* its exact cochlear site-of-lesion could not be determined. Still, it was categorized as pre-synaptic due to its predominant expression in the spiral limbus and ligament [76]. Consequently, 27 nuclear genes were classified as pre-synaptic, one as post-synaptic (*OPA1*), and one as mitochondrial (*LARS2)* along with the mitochondrial genes (Table 1), totalling 286 ears (*N* = 211 subjects) included in the following analysis.

**Fig. 2:**
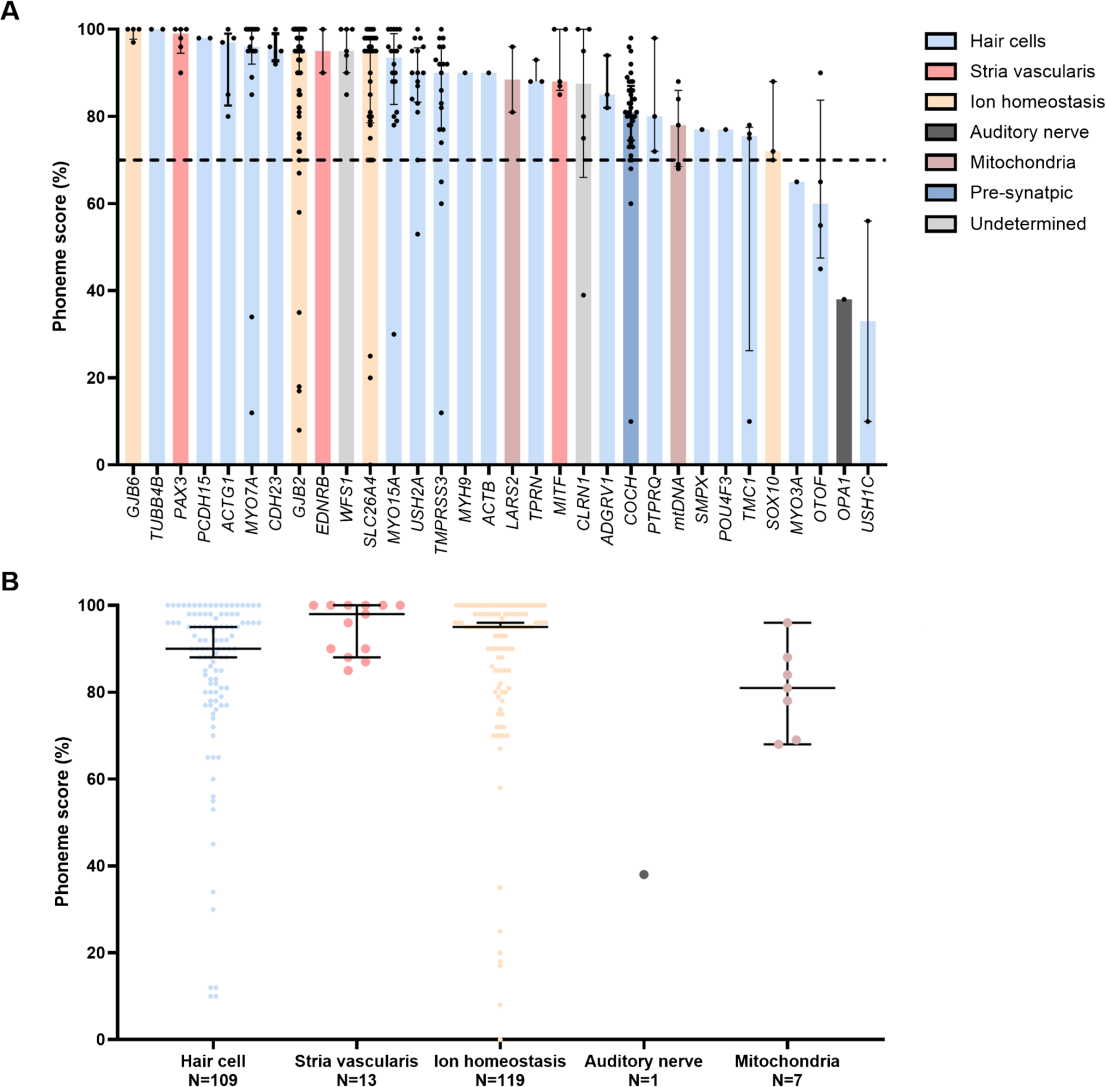
CI outcomes per gene and cochlear site of lesion. **A** Cochlear implant outcomes per gene. Bars show median phoneme scores at 65 dB SPL in quiet, arranged from highest to lowest. Dots indicate individual data points, with the plot representing interquartile ranges. **B** Scatterplots of the last measured phoneme scores 65 dB SPL in quiet, broken down by subgroup. Each plot represents the median with interquartile ranges. Subjects with pathogenic variants in *COCH* (*N* = 37 ears) were excluded due to the gene’s broad expression in the pre-synaptic part of the cochlea. Similarly, subjects with variants in *CLRN1* (*N* = 6 ears) and *WFS1* (*N* = 7 ears) were excluded due to their association with both pre- and post-synaptic pathology

Figure 2A shows CI outcomes per affected gene, with most genes associated with good performance (median phoneme scores > 70%). Poorer outcomes (median phoneme score < 70%) were linked to *MYO3 A* (*N* = 1 ear)*, OTOF* (*N* = 3 ears)*, OPaA1* (*N* = 1 ear)*,* and *USH1 C* (*N* = 2 ears)*. OPA1* is classified as a post-synaptic gene, while *MYO3 A, OTOF,* and *USH1 C* are associated with HC pathologies and are thus classified as pre-synaptic genes.

Figure 2B shows the CI outcomes per cochlear site-of-lesion, demonstrating high median phoneme scores in the HC, stria vascularis, and ion homeostasis groups and a lower median score in the mitochondrial group. The single subject with *OPA1*-associated SNHL (*N* = 1 ear), categorized in the post-synaptic group, had poor outcomes (phoneme score of 38% eight years post-implantation).

Within the pre-synaptic group, no significant difference was found between the stria vascularis and ion homeostasis group (*p* = 0.130). In contrast, the HC group had significantly lower phoneme scores (90% (IQR 80–98)) than the stria vascularis group (98% (IQR 89–100); *p* = 0.020) and the ion homeostasis group (95% (IQR 85–98); *p* = 0.043; Fig. 2B). These differences remained significant after excluding non-users (*N* = 8 ears and *N* = 7 ears for the stria vascularis and ion homeostasis groups, respectively) from the analysis (*p* = 0.041 and *p* = 0.042, respectively). Further analysis showed that the cochlear site-of-lesion did not correlate with CI outcomes within the pre-synaptic group (*R*^2^ = 0.005, *p* = 0.275), even after correcting for confounding factors as listed in Table 4. The lower phoneme scores observed in the HC group compared to the stria vascularis and ion homeostasis groups are primarily due to a higher number of subject-specific factors associated with poorer performance within the HC group (Table 5), combined with the observation that the majority of poorer performers (15 of the 29 individuals) fall within the HC group.

**Table 4:** Correlation analysis with the phoneme score at 65 dB SPL in quiet as the dependent variable

Dependant variable	Last measured phoneme score at 65 dB SPL in quiet			
	Univariate regression analysis			
Variable	*R*^2^	DF	*F*	*p*
Gender	0.017	284	4.862	0.028^+^
Age at implantation	0.082	284	25.488	< 0.001^+^
Self-reported duration of SNHL	0.017	284	4.783	0.030^+^
Duration of CI experience	0.097	284	30.437	< 0.001^+^
HA in ear to be implanted	0.024	284	7.065	0.008^+^
Degree of SNHL pre-implantation	0.007	250	1.679	0.196
Residual hearing	0.001	100	0.150	0.699
Implant type (PME vs LWE)	0.002	284	0.496	0.482
Implant Brand	0.005	284	1.492	0.223
Year of implantation	0.000	284	0.002	0.965
Type of insertion	0.001	284	0.271	0.603
Cochlear site-of-lesion*	0.000	247	0.006	0.938
	Adjusted *R*^2^	DF	*F*	*p*
Model 1**	0.441	54	5.345	< 0.001
Model 2 ***	0.187	235	12.061	< 0.001
Model 3****	0.194	280	14.692	< 0.001

*DF* degree of freedom, *F* F-test, *p* significancy, *SNHL* sensorineural hearing loss, *HA* hearing aids, *PME* peri-modiolar electrode, *LWE* lateral wall electrode

^*^Cochlear site-of-lesion includes hair cells, stria vascularis, ion homeostasis, auditory nerve, and mitochondria

^**^Model 1 includes all variables listed above in Table 3. The variables gender, HA in the ear to be implanted, residual hearing, type of electrode, brand, year of implantation, and cochlear site-of-lesion do not significantly contribute to this model

^***^Model 2 includes the cochlear site-of-lesion and all factors marked with a plus sign (^+^). In this model, the cochlear site-of-lesion did not significantly contribute

^****^Model 3 was conducted using backward selection and includes all factors marked with a plus sign (^+^). All factors are significantly attributed to this model

**Table 5:** Poor cochlear implant performers

Reason for poor performance	Number of ears		Involved genes (*N* ears)	
Prelingual SNHL and implanted during adulthood	8	(26.7%)	*GJB2* *MYO7 A* *TMC1* *USH1 C*	(4) (2) (1) (1)
Struggling to adjust to the sound of the second sequentially implanted CI	6	(20.0%)	*GJB2* *MYO15 A* *OTOF* *SLC26 A4* *USH1 C* *USH2 A*	(1) (1) (1) (1) (1) (1)
Post-lingual SNHL with an older age at implantation and a prolonged period without sufficient auditory stimulation prior to implantation	6	(20.0%)	*COCH* mtDNA *MYO3 A* *TMPRSS3*	(2) (2) (1) (1)
Minimal encouragement from home or school for deaf to use the CI	4	(13.3%)	*GJB2* *OTOF* *SLC26 A4*	(1) (2) (1)
High-frequency SNHL with sufficient residual hearing in the lower frequencies post-implantation	2	(6.7%)	*TMPRSS3*	(2)
Device failure	2	(6.7%)	*COCH* *SLC26 A4*	(1) (1)
Auditory nerve hypoplasia or neuropathy	2	(6.7%)	*CLRN1* *OPA1*	(1) (1)

*SNHL* sensorineural hearing loss, *CI* cochlear implant, *mtDNA* mitochondrial DNA

Comparison between pre-synaptic and post-synaptic groups was impossible due to the limited number of post-synaptic subjects (*N* = 1). The pre-synaptic group did not demonstrate significantly better outcomes than the mitochondrial group (*p* = 0.055).

### Factors Contributing to the Variance in Cochlear Implantation Outcomes

We initially performed univariate regression analyses with the included 286 ears (*N* = 211 subjects) to identify factors significantly correlated with CI outcomes (Table 4). Five variables showed significant correlations, with duration of CI experience and age at implantation demonstrating the most substantial effects (*R*^2^ = 0.097, *p* < 0.001; and *R*^2^ = 0.082, *p* < 0.001, respectively). The cochlear site-of-lesion did not correlate with CI outcomes (*R*^2^ = 0.000, *p* = 0.938).

Additionally, multivariate regression analyses revealed that all factors listed in Table 4 (model 1) collectively accounted for 44.1% of the variance in phoneme scores (*p* < 0.001), although not all factors significantly contributed. A prediction model using backward selection was constructed in which all factors significantly contributed, including sex, age at implantation, self-reported duration of SNHL, CI experience, and HA in the ear to be implanted. This second model accounted for 19.0% of the variance in CI outcomes (*p* < 0.001).

### Poorer Cochlear Implant Performers

Among the total study population, 29 individuals (13.4%; *N* = 30 ears (10.0%)) were poorer performers, defined as those with a last measured phoneme score < 70%. This resulted in non- or limited CI use in 14 individuals (6.5%; 15 ears (5.0%)). Among these individuals, 14 affected genes were identified across three groups (pre-/post-synaptic and mitochondrial). Multiple factors contributing to lower phoneme scores were identified, with poorer performance most frequently observed in individuals with prelingual SNHL and implantation in adulthood (26.7%) and those with sequential implanted CIs struggling to adapt to the sound of the second implant (20.0%). Furthermore, poorer performance was also common in individuals with post-lingual SNHL who underwent cochlear implantation at an older age after experiencing a prolonged period without adequate auditory stimulation (20.0%). Additional factors that contributed less are listed in Table 4.

## Discussion

In this study of the largest cohort of genotyped CI recipients to date (*N* = 220), we aimed to evaluate CI outcomes and their correlation with affected genes and cochlear site-of-lesion. In line with previous studies involving collectively 334 genotyped CI recipients [11–17], our findings identified *GJB2, COCH, SLC26 A4, TMPRSS3*, and *MYO7 A* as the most frequently affected genes in genetic SNHL (Supplementary Table 1). Notably, in our Dutch cohort, a higher prevalence of causative variants in *COCH* was observed compared to the previous Asian, American, and German cohorts. This is attributable to demographic factors, as a specific variant in *COCH* is inherited from a common ancestor and is predominantly present in the southern parts of the Netherlands and in Belgium [77].

### CI Outcomes in Genotyped CI Recipients

The first aim of our study was to evaluate CI outcomes in genotyped CI recipients. Our analysis of 220 recipients (*N* = 299 ears) demonstrated generally excellent short- and long-term outcomes, with a median phoneme score of 90% (IQR 80–98) at the last follow-up. Notably, subjects who received their first CI before the age of seven years had significantly higher phoneme scores (96% compared to 82%) and showed less variability than those who were implanted at a later age. These findings are consistent with previous studies [13, 16, 19–21], underscoring the importance of early implantation, which benefits from the greater plasticity of the central auditory system before the age of seven [70, 71]. Moreover, the present cohort also showed a higher median score than the cohort in the study by Tropitzsch et al., who reported a median word recognition score of 70% [15]. This is likely due to a longer self-reported duration of SNHL and an older age at implantation than in our cohort. Both factors have been associated with poorer outcomes [3].

### CI Outcomes per Gene

Our second aim was to evaluate CI outcomes per gene. While most pathogenic variants in nuclear and mitochondrial genes led to beneficial outcomes, i.e., with phoneme scores in quiet of > 70%, four genes (*MYO3 A, OTOF, OPA1,* and *USH1 C)* were linked to poorer performance. However, each of these four groups included only a small number of subjects. *MYO3 A, OTOF,* and *USH1 C* are all associated with HC pathology [78–80], while *OPA1* is associated with auditory neuropathy [81]. CI outcomes in *MYO3 A-*associated SNHL are not well-documented, with only one well-performing [16] and one poorly performing case reported [82]. CI outcomes for *OTOF* and *OPA1-*associated SNHL are generally beneficial [16, 19, 83–86]. While specific CI outcomes for Usher syndrome type 1 C are lacking, overall outcomes in Usher syndrome type 1 are positive when CIs are implanted early [33, 87–89].

The previously reported favorable CI outcomes for *OTOF, OPA1*, and *USH1 C* contradict the poorer outcomes observed in our study subjects. The identification of subject-specific factors in cases with causative pathogenic variants in *MYO3 A, OTOF,* and *USH1 C* (Table 5) suggests that these factors, rather than gene-specific ones, play a critical role in this discrepancy. These subject-specific factors include pre-lingual SNHL with cochlear implantation in adulthood, older age at implantation with a prolonged period of auditory deprivation, and minimal support system encouragement. This shows that the poorer performance observed among these subjects was independent of the causative genetic defects.

In contrast, we did not find subject-specific factors in the subject with *OPA1-*associated SNHL, who had stable phoneme scores of 70–80% for the initial five years post-implantation, which gradually declined to 38% by eight years post-implantation. This subject received a perimodiolar electrode array, and no implant failure was observed. Since cochlear nerve fibers in *OPA1-*associated SNHL likely degenerate over time [83], a decline in CI performance is anticipated. Moreover, *OPA1* is associated not only with SNHL but also with optic atrophy and peripheral neuropathy, leading to progressive vision loss, sensory loss, and muscle weakness [90, 91]. This underscores the progressive peripheral nerve degeneration caused by *OPA1* variants and highlights the need for future research to focus on long-term CI outcomes in *OPA1-*associated SNHL.

### CI Outcomes per Cochlear Site-of-Lesion

The third aim of this study was to correlate CI outcomes with the cochlear site-of-lesion, for which we categorized affected genes into five groups: HC, stria vascularis, ion homeostasis, cochlear nerve, and mitochondria. The cochlear site-of-lesion did not account for CI outcome variability (*R*^2^ = 0.000, *p* = 0.938), and we found no significant differences in outcomes between the pre-synaptic and mitochondrial groups. Most affected genes were classified as pre-synaptic (*N* = 27 genes), while only one gene was classified as post-synaptic, making a reliable comparison of pre-and post-synaptic cases impossible. Note, however, that we excluded *CLRN1* and *WFS1* from our analysis as these are associated with both pre-and post-synaptic pathology [35, 70, 71].

In contrast, Tropitzsch et al. observed poorer CI performance in subjects with variants affecting the cochlea’s neural component [16]. However, when applying their classification system to our dataset, we found no significant difference in CI outcomes between the pre- and post-synaptic groups (Supplementary Fig. 1). This discrepancy can be explained by the fact that, among the eight genes they classified as post-synaptic (*TMPRSS3*, *EDNRB*, *SOX10*, *WFS1*, *PAX3*, *EYA4*, *TFAP2 A*, and *DIAPH1*), our dataset only included subjects with pathogenic variants in *TMPRSS3*, *WFS1*, and *PAX3*, all of whom generally had good CI outcomes (Fig. 2A). Furthermore, in previously published studies, we specifically evaluated CI outcomes in *TMPRSS3-* and *WFS1-*associated SNHL and concluded that, given their favorable short- and long-term CI outcomes, these genes are more likely associated with pre-synaptic rather than post-synaptic pathology [32, 75].

However, both our study and Troptizsch et al. faced challenges in determining the cochlear site-of-lesion. Tropitzsch et al. based the cochlear site-of-lesion on gene expression levels [16]. Instead, we first evaluated protein function and considered expression patterns if protein function was unknown. This resulted in differing classifications for certain genes. For example, Tropitzsch et al. categorized eight genes (*TMPRSS*, *EDNR*, *SOX1*, *WFS1*, *PAX3*, *EYA4*, *TFAP2 A*, and *DIAPH1*) in the neural group. In contrast, our method classified seven of these genes (all except *TFAP2 A*) as (also) linked to pre-synaptic pathology [74, 92–98]. Consequently, also in the study of Troptizsch et al., only one individual had pathogenic variants in a gene solely associated with post-synaptic pathology.

Both methods have limitations, as predominant expression in a certain cochlear structure does not necessarily mean it is most affected. Furthermore, protein function and expression levels are typically assessed in mouse models, which may not fully represent those in humans. Additionally, some genes have broad expression patterns or are associated with both pre- and post-synaptic pathology, complicating their classification. This highlights the need for caution in interpreting cochlear site-of-lesion classifications and emphasizes the necessity of further research. Future studies should focus on understanding protein function and (sensori)neural health integrity in genotyped individuals to better identify the cochlear site of lesion. Possible methods to consider are utilizing inner ear organoids to evaluate expression patterns [99, 100], or employing electrocochleography to assess (sensori)neural health [101, 102].

Although there is currently no strong evidence supporting the hypothesis that post-synaptic genetic etiologies are correlated with poorer CI outcomes, other studies suggest that individuals with SNHL due to post-synaptic pathology tend to have less favorable CI outcomes. For example, suboptimal outcomes have been observed in cases of lower SGN survival [103], cochlear nerve deficiency [84], cochlear nerve hypoplasia [104], and vestibular schwannoma [105, 106]. In contrast, generally positive CI outcomes are reported for etiologies associated with pre-synaptic pathologies. For instance, individuals with sudden deafness [107] or SNHL caused by ototoxic drugs [108], both linked to hair cell pathology [109, 110], show successful CI outcomes. This underlines the importance of further research into the correlation between CI outcomes and the cochlear site-of-lesion.

### Factors of Influence on CI Outcomes in Genotyped CI Recipients

While the cochlear site-of-lesion did not significantly impact CI outcomes, other known factors did. Better outcomes were linked to younger age at implantation, longer duration of CI experience, use of an HA in the ear to be implanted, shorter self-reported duration of SNHL, and female sex. This is in line with previous research that has consistently linked these factors to better CI outcomes, except for sex [3, 4, 16]. Better outcomes in our cohort compared to other cohorts may be related to the inclusion of pediatric subjects in our study because in pediatric populations, younger age at implantation is particularly associated with better outcomes [87, 111, 112].

To further assess the impact of these factors, we constructed a linear model of CI outcome with the factors sex, CI experience, age at implantation, HA use, and duration of SNHL. This overall model explained 19% of the total variance, similar to the model by Lazard et al., that accounted for 22% of the variance in outcomes among adult CI recipients [4]. Poorer performance in our study was most frequently observed in subjects with pre-lingual SNHL who received a CI during adulthood (Table 5), a well-known factor associated with poor performance [113]. Post-lingual SNHL with older age at implantation and prolonged auditory deprivation before implantation were also frequently linked to poorer outcomes, consistent with previous studies [4, 114]. Additionally, poorer performance was noted in subjects who received a second CI sequentially but struggled to adapt to its sound, often leading to non-use.

Less frequently observed factors associated with poorer performance included device failure (*N* = 2), minimal encouragement from the supporting system to use the CI (*N* = 4), and ski-slope hearing loss with functional residual hearing in the lower frequencies post-implantation (*N* = 2). In the latter case, subjects had difficulty adapting to the CI sound or experienced limited benefits, resulting in restricted or non-use of the CI.

Finally, neural factors, such as cochlear nerve hypoplasia or auditory neuropathy, as was observed in the subjects with pathogenic variants in *CLRN1* and *OPA1*, respectively, may result in poorer outcomes. In the case of cochlear nerve hypoplasia, the reduced number of nerve fibers could impair the transmission of electrically evoked signals from the implant to the brain. This aligns with previous studies showing poorer CI outcomes in recipients with cochlear nerve deficiencies than those with normal cochlear nerves [115–118].

### Conclusion and Clinical Implications

This study presents a comprehensive evaluation of the currently largest cohort of genotyped CI recipients, which shows that most individuals achieve excellent short- and long-term CI outcomes. The variability in speech perception was not correlated with the cochlear site-of-lesion but likely stemmed from subject-specific rather than underlying genetic defects. Our model, which includes sex, duration of CI experience, age at implantation, HA use, and duration of SNHL, explained 19.0% of this variance. The present findings suggest that most individuals with hereditary SNHL benefit from cochlear implantation, as most affected genes affect the pre-synaptic part of the cochlea. Poorer outcomes are anticipated in subjects who are older during implantation, have a longer duration of SNHL, and stopped using HA for a long time prior to implantation. Pre-implantation counselling should highlight the potentially poorer outcomes in individuals with SNHL linked to genes affecting the post-synaptic cochlea exclusively. However, further research is required to assess the correlation between cochlear site-of-lesion and CI outcomes.

## Supplementary Information

Below is the link to the electronic supplementary material.

Supplementary file1 (DOCX 74.9 KB)

Supplementary file2 (DOCX 100 KB)

## Data Availability

The datasets used and analyzed during the current study are available from the corresponding author on reasonable request.

## Code Availability

The codes used analyzing the date during the current study are available form the corresponding author on reasonable request.

## Declarations

### AI Use

ChatGPT ((https://chat.openai.com/) was used between September 2024 and January 2025 to refine the academic English in the introduction, methods, and discussion sections of this article. The authors take full responsibility for the accuracy and integrity of the content. AI was not used to generate data or figures.
