# Supplementary material for: Cochlear Implantation Outcomes in Genotyped Subjects with Sensorineural Hearing Loss
Source: J Assoc Res Otolaryngol. 2025 Apr 23;26(3):331–48. doi: 10.1007/s10162-025-00987-0 (PMC12133674; doi:10.1007/s10162-025-00987-0)
Supplement: Supplementary file 1 — Supplementary file1 (DOCX 74.9 KB) [file 10162_2025_987_MOESM1_ESM.docx]

**Supplementary Table 1. Gene distribution**

| Gene | Wu et al. (2011)  Taiwan (2)  N(%) | | Park et al. (2014)  Korea (3)  N(%) | | Lee et al. (2020) Korea (4)  N(%) | | Yoshimura et al. (2020) Japan (5)  N(%) | | Seligman et al. (2022) America (6)  N(%) | | Tropitzsch et al. (2023)  Duitsland (7)  N(%) | | Total previous studies*  N(%) | | This study  The Netherlands  N(%) | | Total all studies  N(%) | |
| --- | --- | --- | --- | --- | --- | --- | --- | --- | --- | --- | --- | --- | --- | --- | --- | --- | --- | --- |
| *ACTB* | 0 | 0.00 | 0 | 0.00 | 0 | 0.00 | 0 | 0.00 | 0 | 0.00 | 0 | 0.00 | 0 | 0,00 | 1 | 0,37 | 1 | 0,16 |
| *ACTG1* | 0 | 0.00 | 1 | 1,96 | 1 | 4,76 | 2 | 10,53 | 2 | 1,52 | 1 | 1,35 | 7 | 2,10 | 6 | 2,20 | 13 | 2,14 |
| *ADGRV1* | 0 | 0.00 | 0 | 0.00 | 0 | 0.00 | 0 | 0.00 | 0 | 0.00 | 0 | 0.00 | 0 | 0,00 | 2 | 0,73 | 2 | 0,33 |
| *ALMS1* | 0 | 0.00 | 0 | 0.00 | 0 | 0.00 | 0 | 0.00 | 1 | 0,76 | 0 | 0.00 | 1 | 0,30 | 0 | 0,00 | 1 | 0,16 |
| *ATP1A3* | 0 | 0.00 | 0 | 0.00 | 2 | 9,52 | 0 | 0.00 | 0 | 0.00 | 0 | 0.00 | 2 | 0,60 | 0 | 0,00 | 2 | 0,33 |
| *CDH23* | 0 | 0.00 | 3 | 5,88 | 2 | 9,52 | 5 | 26,32 | 1 | 0,76 | 2 | 2,7 | 13 | 3,89 | 4 | 1,47 | 17 | 2,80 |
| *CDH7* | 0 | 0.00 | 7 | 13,73 | 0 | 0.00 | 0 | 0.00 | 0 | 0.00 | 0 | 0.00 | 7 | 2,10 | 6 | 2,20 | 13 | 2,14 |
| *CLDN14* | 0 | 0.00 | 0 | 0.00 | 0 | 0.00 | 1 | 5,26 | 1 | 0,76 | 0 | 0.00 | 2 | 0,60 | 0 | 0,00 | 2 | 0,33 |
| *CLRN1* | 0 | 0.00 | 0 | 0.00 | 0 | 0.00 | 0 | 0.00 | 0 | 0.00 | 0 | 0.00 | 0 | 0,00 | 4 | 1,47 | 4 | 0,66 |
| *COCH* | 0 | 0.00 | 0 | 0.00 | 2 | 9,52 | 0 | 0.00 | 3 | 2,27 | 1 | 1,35 | 6 | 1,80 | 34 | 12,45 | 40 | 6,58 |
| *COL11A1* | 0 | 0.00 | 0 | 0.00 | 0 | 0.00 | 0 | 0.00 | 4 | 3,03 | 1 | 1,35 | 5 | 1,50 | 0 | 0,00 | 5 | 0,82 |
| *COL11A2* | 0 | 0.00 | 0 | 0.00 | 0 | 0.00 | 0 | 0.00 | 1 | 0,76 | 0 | 0.00 | 1 | 0,30 | 0 | 0,00 | 1 | 0,16 |
| *COL2A1* | 0 | 0.00 | 0 | 0.00 | 0 | 0.00 | 0 | 0.00 | 1 | 0,76 | 1 | 1,35 | 2 | 0,60 | 0 | 0,00 | 2 | 0,33 |
| *COL4A3* | 0 | 0.00 | 0 | 0.00 | 0 | 0.00 | 0 | 0.00 | 0 | 0.00 | 3 | 4,05 | 3 | 0,90 | 0 | 0,00 | 3 | 0,49 |
| *COL4A5* | 0 | 0.00 | 0 | 0.00 | 0 | 0.00 | 0 | 0.00 | 1 | 0,76 | 0 | 0.00 | 1 | 0,30 | 0 | 0,00 | 1 | 0,16 |
| *DIAPH1* | 0 | 0.00 | 0 | 0.00 | 0 | 0.00 | 0 | 0.00 | 2 | 1,52 | 1 | 1,35 | 3 | 0,90 | 1 | 0,37 | 4 | 0,66 |
| *EDNRB* | 0 | 0.00 | 0 | 0.00 | 0 | 0.00 | 0 | 0.00 | 0 | 0.00 | 1 | 1,35 | 1 | 0,30 | 1 | 0,37 | 2 | 0,33 |
| *EYA1* | 0 | 0.00 | 0 | 0.00 | 0 | 0.00 | 0 | 0.00 | 0 | 0.00 | 0 | 0.00 | 0 | 0,00 | 1 | 0,37 | 1 | 0,16 |
| *EYA4* | 0 | 0.00 | 0 | 0.00 | 0 | 0.00 | 0 | 0.00 | 0 | 0.00 | 1 | 1,35 | 1 | 0,30 | 0 | 0,00 | 1 | 0,16 |
| *FDXR* | 0 | 0.00 | 0 | 0.00 | 0 | 0.00 | 0 | 0.00 | 0 | 0.00 | 0 | 0.00 | 0 | 0,00 | 1 | 0,37 | 1 | 0,16 |
| *GJB2* | 13 | 35,14 | 10 | 19,61 | 1 | 4,76 | 0 | 0.00 | 20 | 15,15 | 14 | 18,92 | 58 | 17,37 | 55 | 20,15 | 113 | 18,59 |
| *GJB6* | 0 | 0.00 | 0 | 0.00 | 0 | 0.00 | 0 | 0.00 | 0 | 0.00 | 0 | 0.00 | 0 | 0,00 | 2 | 0,73 | 2 | 0,33 |
| *GPR98* | 0 | 0.00 | 0 | 0.00 | 0 | 0.00 | 0 | 0.00 | 2 | 1,52 | 0 | 0.00 | 2 | 0,60 | 0 | 0,00 | 2 | 0,33 |
| *ILDR1* | 0 | 0.00 | 0 | 0.00 | 1 | 4,76 | 0 | 0.00 | 0 | 0.00 | 0 | 0.00 | 1 | 0,30 | 0 | 0,00 | 1 | 0,16 |
| *KARS* | 0 | 0.00 | 0 | 0.00 | 0 | 0.00 | 0 | 0.00 | 0 | 0.00 | 0 | 0.00 | 0 | 0,00 | 1 | 0,37 | 1 | 0,16 |
| *KCNE1* | 0 | 0.00 | 1 | 1,96 | 0 | 0.00 | 0 | 0.00 | 0 | 0.00 | 1 | 1,35 | 2 | 0,60 | 0 | 0,00 | 2 | 0,33 |
| *KCNQ4* | 0 | 0.00 | 0 | 0.00 | 0 | 0.00 | 0 | 0.00 | 2 | 1,52 | 0 | 0.00 | 2 | 0,60 | 0 | 0,00 | 2 | 0,33 |
| *KITLG* | 0 | 0.00 | 0 | 0.00 | 0 | 0.00 | 0 | 0.00 | 0 | 0.00 | 0 | 0.00 | 0 | 0,00 | 1 | 0,37 | 1 | 0,16 |
| *KNCQ1* | 0 | 0.00 | 0 | 0.00 | 0 | 0.00 | 0 | 0.00 | 1 | 0,76 | 0 | 0.00 | 1 | 0,30 | 0 | 0,00 | 1 | 0,16 |
| *LARS2* | 0 | 0.00 | 0 | 0.00 | 0 | 0.00 | 0 | 0.00 | 0 | 0.00 | 0 | 0.00 | 0 | 0,00 | 1 | 0,37 | 1 | 0,16 |
| *LHFPL5* | 0 | 0.00 | 0 | 0.00 | 0 | 0.00 | 0 | 0.00 | 1 | 0,76 | 1 | 1,35 | 2 | 0,60 | 0 | 0,00 | 2 | 0,33 |
| *LOXHD1* | 0 | 0.00 | 0 | 0.00 | 0 | 0.00 | 1 | 5,26 | 4 | 3,03 | 3 | 4,05 | 8 | 2,40 | 0 | 0,00 | 8 | 1,32 |
| *MARVELD2* | 0 | 0.00 | 0 | 0.00 | 0 | 0.00 | 0 | 0.00 | 0 | 0.00 | 2 | 2,7 | 2 | 0,60 | 0 | 0,00 | 2 | 0,33 |
| *MITF* | 0 | 0.00 | 0 | 0.00 | 0 | 0.00 | 0 | 0.00 | 0 | 0.00 | 2 | 2,7 | 2 | 0,60 | 7 | 2,56 | 9 | 1,48 |
| *MT-TL1* | 0 | 0.00 | 0 | 0.00 | 0 | 0.00 | 0 | 0.00 | 0 | 0.00 | 1 | 1,35 | 1 | 0,30 | 0 | 0,00 | 1 | 0,16 |
| *mtDNA* | 1 | 2,7 | 0 | 0.00 | 0 | 0.00 | 2 | 10,53 | 7 | 5,3 | 0 | 0.00 | 10 | 2,99 | 6 | 2,20 | 16 | 2,63 |
| *MYH14* | 0 | 0.00 | 0 | 0.00 | 0 | 0.00 | 0 | 0.00 | 3 | 2,27 | 2 | 2,7 | 5 | 1,50 | 0 | 0,00 | 5 | 0,82 |
| *MYH9* | 0 | 0.00 | 0 | 0.00 | 0 | 0.00 | 0 | 0.00 | 1 | 0,76 | 0 | 0.00 | 1 | 0,30 | 1 | 0,36 | 2 | 0,33 |
| *MYO15A* | 0 | 0.00 | 2 | 3,92 | 1 | 4,76 | 2 | 10,53 | 5 | 3,79 | 5 | 6,76 | 15 | 4,49 | 15 | 5,49 | 30 | 4,93 |
| *MYO3A* | 0 | 0.00 | 1 | 1,96 | 0 | 0.00 | 0 | 0.00 | 0 | 0.00 | 1 | 1,35 | 2 | 0,60 | 1 | 0,36 | 3 | 0,49 |
| *MYO6* | 0 | 0.00 | 0 | 0.00 | 0 | 0.00 | 0 | 0.00 | 4 | 3,03 | 2 | 2,7 | 6 | 1,80 | 1 | 0,36 | 7 | 1,15 |
| *MYO7A* | 0 | 0.00 | 2 | 3,92 | 1 | 4,76 | 2 | 10,53 | 9 | 6,82 | 3 | 4,05 | 17 | 5,09 | 15 | 5,47 | 32 | 5,26 |
| *NF2* | 0 | 0.00 | 0 | 0.00 | 1 | 4,76 | 0 | 0.00 | 0 | 0.00 | 0 | 0.00 | 1 | 0,30 | 0 | 0,00 | 1 | 0,16 |
| *NLRP3* | 0 | 0.00 | 0 | 0.00 | 1 | 4,76 | 0 | 0.00 | 0 | 0.00 | 0 | 0.00 | 1 | 0,30 | 0 | 0,00 | 1 | 0,16 |
| *OPA1* | 0 | 0.00 | 0 | 0.00 | 0 | 0.00 | 0 | 0.00 | 1 | 0,76 | 0 | 0.00 | 1 | 0,30 | 1 | 0,31 | 2 | 0,33 |
| *OSBPL2* | 0 | 0.00 | 0 | 0.00 | 0 | 0.00 | 0 | 0.00 | 1 | 0,76 | 0 | 0.00 | 1 | 0,30 | 0 | 0,00 | 1 | 0,16 |
| *OTOF* | 2 | 5,41 | 1 | 1,96 | 0 | 0.00 | 0 | 0.00 | 2 | 1,52 | 1 | 1,35 | 6 | 1,80 | 5 | 1,82 | 11 | 1,81 |
| *OTOG* | 0 | 0.00 | 0 | 0.00 | 0 | 0.00 | 0 | 0.00 | 2 | 1,52 | 0 | 0.00 | 2 | 0,60 | 0 | 0,00 | 2 | 0,33 |
| *P2RX2* | 0 | 0.00 | 0 | 0.00 | 0 | 0.00 | 0 | 0.00 | 1 | 0,76 | 0 | 0.00 | 1 | 0,30 | 0 | 0,00 | 1 | 0,16 |
| *PAX3* | 0 | 0.00 | 0 | 0.00 | 0 | 0.00 | 0 | 0.00 | 1 | 0,76 | 2 | 2,7 | 3 | 0,90 | 5 | 1,82 | 8 | 1,32 |
| *PCDH15* | 0 | 0.00 | 1 | 1,96 | 0 | 0.00 | 0 | 0.00 | 0 | 0.00 | 0 | 0.00 | 1 | 0,30 | 1 | 0,36 | 2 | 0,33 |
| *PDZ7* | 0 | 0.00 | 0 | 0.00 | 0 | 0.00 | 0 | 0.00 | 1 | 0,76 | 0 | 0.00 | 1 | 0,30 | 0 | 0,00 | 1 | 0,16 |
| *POLR1D* | 0 | 0.00 | 0 | 0.00 | 0 | 0.00 | 0 | 0.00 | 1 | 0,76 | 0 | 0.00 | 1 | 0,30 | 0 | 0,00 | 1 | 0,16 |
| *POU3F4* | 0 | 0.00 | 5 | 9,8 | 0 | 0.00 | 0 | 0.00 | 1 | 0,76 | 1 | 1,35 | 7 | 2,10 | 2 | 0,73 | 9 | 1,48 |
| *POU4F3* | 0 | 0.00 | 0 | 0.00 | 0 | 0.00 | 0 | 0.00 | 6 | 4,55 | 1 | 1,35 | 7 | 2,10 | 0 | 0,00 | 7 | 1,15 |
| *PRPS1* | 0 | 0.00 | 0 | 0.00 | 0 | 0.00 | 0 | 0.00 | 0 | 0.00 | 0 | 0.00 | 0 | 0,00 | 1 | 0,36 | 1 | 0,16 |
| *PTPN11* | 0 | 0.00 | 0 | 0.00 | 0 | 0.00 | 0 | 0.00 | 0 | 0.00 | 0 | 0.00 | 0 | 0,00 | 4 | 1,46 | 4 | 0,66 |
| *PTPRQ* | 0 | 0.00 | 0 | 0.00 | 0 | 0.00 | 0 | 0.00 | 0 | 0.00 | 1 | 1,35 | 1 | 0,30 | 2 | 0,73 | 3 | 0,49 |
| *RRM2B* | 0 | 0.00 | 0 | 0.00 | 0 | 0.00 | 0 | 0.00 | 0 | 0.00 | 1 | 1,35 | 1 | 0,30 | 1 | 0,36 | 2 | 0,33 |
| *SCN7A* | 0 | 0.00 | 0 | 0.00 | 0 | 0.00 | 0 | 0.00 | 2 | 1,52 | 0 | 0.00 | 2 | 0,60 | 0 | 0,00 | 2 | 0,33 |
| *SERPNB6* | 0 | 0.00 | 0 | 0.00 | 1 | 4,76 | 0 | 0.00 | 0 | 0.00 | 0 | 0.00 | 1 | 0,30 | 0 | 0,00 | 1 | 0,16 |
| *SIX1* | 0 | 0.00 | 0 | 0.00 | 0 | 0.00 | 0 | 0.00 | 1 | 0,76 | 0 | 0.00 | 1 | 0,30 | 0 | 0,00 | 1 | 0,16 |
| *SLC17A8* | 0 | 0.00 | 0 | 0.00 | 0 | 0.00 | 0 | 0.00 | 1 | 0,76 | 0 | 0.00 | 1 | 0,30 | 0 | 0,00 | 1 | 0,16 |
| *SLC26A4* | 21 | 56,76 | 13 | 25,49 | 2 | 9,52 | 2 | 10,53 | 10 | 7,58 | 3 | 4,05 | 51 | 15,27 | 30 | 10,95 | 81 | 13,32 |
| *SMPX* | 0 | 0.00 | 0 | 0.00 | 0 | 0.00 | 0 | 0.00 | 1 | 0,76 | 2 | 2,7 | 3 | 0,90 | 1 | 0,36 | 4 | 0,66 |
| *SOX10* | 0 | 0.00 | 0 | 0.00 | 0 | 0.00 | 0 | 0.00 | 0 | 0.00 | 1 | 1,35 | 1 | 0,30 | 3 | 1,09 | 4 | 0,66 |
| *TBC1D24* | 0 | 0.00 | 0 | 0.00 | 0 | 0.00 | 0 | 0.00 | 1 | 0,76 | 0 | 0.00 | 1 | 0,30 | 0 | 0,00 | 1 | 0,16 |
| *TECTA* | 0 | 0.00 | 0 | 0.00 | 0 | 0.00 | 0 | 0.00 | 4 | 3,03 | 0 | 0.00 | 4 | 1,20 | 0 | 0,00 | 4 | 0,66 |
| *TFAP2A* | 0 | 0.00 | 0 | 0.00 | 0 | 0.00 | 0 | 0.00 | 0 | 0.00 | 1 | 1,35 | 1 | 0,30 | 0 | 0,00 | 1 | 0,16 |
| *TJP2* | 0 | 0.00 | 0 | 0.00 | 0 | 0.00 | 0 | 0.00 | 1 | 0,76 | 0 | 0.00 | 1 | 0,30 | 0 | 0,00 | 1 | 0,16 |
| *TMC1* | 0 | 0.00 | 0 | 0.00 | 3 | 14,29 | 0 | 0.00 | 3 | 2,27 | 0 | 0.00 | 6 | 1,80 | 3 | 1,09 | 9 | 1,48 |
| *TMIE* | 0 | 0.00 | 0 | 0.00 | 0 | 0.00 | 0 | 0.00 | 0 | 0.00 | 1 | 1,35 | 1 | 0,30 | 0 | 0,00 | 1 | 0,16 |
| *TMPRSS3* | 0 | 0.00 | 3 | 5,88 | 2 | 9,52 | 2 | 10,53 | 13 | 9,85 | 6 | 8,11 | 26 | 7,78 | 20 | 7,30 | 46 | 7,57 |
| *TPRN* | 0 | 0.00 | 0 | 0.00 | 0 | 0.00 | 0 | 0.00 | 0 | 0.00 | 0 | 0.00 | 0 | 0,00 | 3 | 1,09 | 3 | 0,49 |
| *TRIOBP* | 0 | 0.00 | 0 | 0.00 | 0 | 0.00 | 0 | 0.00 | 1 | 0,76 | 0 | 0.00 | 1 | 0,30 | 0 | 0,00 | 1 | 0,16 |
| *TUBB4B* | 0 | 0.00 | 0 | 0.00 | 0 | 0.00 | 0 | 0.00 | 0 | 0.00 | 0 | 0.00 | 0 | 0,00 | 1 | 0,36 | 1 | 0,16 |
| *USH1C* | 0 | 0.00 | 0 | 0.00 | 0 | 0.00 | 0 | 0.00 | 0 | 0.00 | 0 | 0.00 | 0 | 0,00 | 3 | 1,09 | 3 | 0,49 |
| *USH2A* | 0 | 0.00 | 1 | 1,96 | 0 | 0.00 | 0 | 0.00 | 1 | 0,76 | 3 | 4,05 | 5 | 1,50 | 15 | 5,47 | 20 | 3,29 |
| *WFS1* | 0 | 0.00 | 0 | 0.00 | 0 | 0.00 | 0 | 0.00 | 0 | 0.00 | 3 | 4,05 | 3 | 0,90 | 6 | 2,19 | 9 | 1,48 |
| Total | 37 | 100,00 | 51 | 100,00 | 21 | 100,00 | 19 | 100,00 | 132 | 100,00 | 74 | 100,00 | 334 | 100,00 | 274 | 100,00 | 608 | 100,00 |

* Data from the study of Miyagawa et al. (1) could not be included in this overview, because the prevalence of identified affected genes was reported as percentage, and the specific genes affected within the Waardenburg syndrome group were not specified.

**References supplementary table 1.**

1. Miyagawa M, Nishio SY, Usami S. A Comprehensive Study on the Etiology of Patients Receiving Cochlear Implantation With Special Emphasis on Genetic Epidemiology. Otol Neurotol. 2016;37(2):e126-34.

2. Wu CC, Liu TC, Wang SH, Hsu CJ, Wu CM. Genetic characteristics in children with cochlear implants and the corresponding auditory performance. Laryngoscope. 2011;121(6):1287-93.

3. Park JH, Kim NK, Kim AR, Rhee J, Oh SH, Koo JW, et al. Exploration of molecular genetic etiology for Korean cochlear implantees with severe to profound hearing loss and its implication. Orphanet J Rare Dis. 2014;9:167.

4. Lee SY, Shim YJ, Han JH, Song JJ, Koo JW, Oh SH, et al. The molecular etiology of deafness and auditory performance in the postlingually deafened cochlear implantees. Sci Rep. 2020;10(1):5768.

5. Yoshimura H, Moteki H, Nishio SY, Miyajima H, Miyagawa M, Usami SI. Genetic testing has the potential to impact hearing preservation following cochlear implantation. Acta Otolaryngol. 2020;140(6):438-44.

6. Seligman KL, Shearer AE, Frees K, Nishimura C, Kolbe D, Dunn C, et al. Genetic Causes of Hearing Loss in a Large Cohort of Cochlear Implant Recipients. Otolaryngol Head Neck Surg. 2022;166(4):734-7.

7. Tropitzsch A, Schade-Mann T, Gamerdinger P, Dofek S, Schulte B, Schulze M, et al. Variability in Cochlear Implantation Outcomes in a Large German Cohort With a Genetic Etiology of Hearing Loss. Ear Hear. 2023.
