# Supplementary material for: Cochlear Implantation Outcomes in Genotyped Subjects with Sensorineural Hearing Loss
Source: J Assoc Res Otolaryngol. 2025 Apr 23;26(3):331–48. doi: 10.1007/s10162-025-00987-0 (PMC12133674; doi:10.1007/s10162-025-00987-0)
Supplement: Supplementary file 2 — Supplementary file2 (DOCX 100 KB) [file 10162_2025_987_MOESM2_ESM.docx]

**Supplementary Figure 1.** **CI outcomes per cochlear site-of-lesion according to Tropitzsch et al.’s classification system**


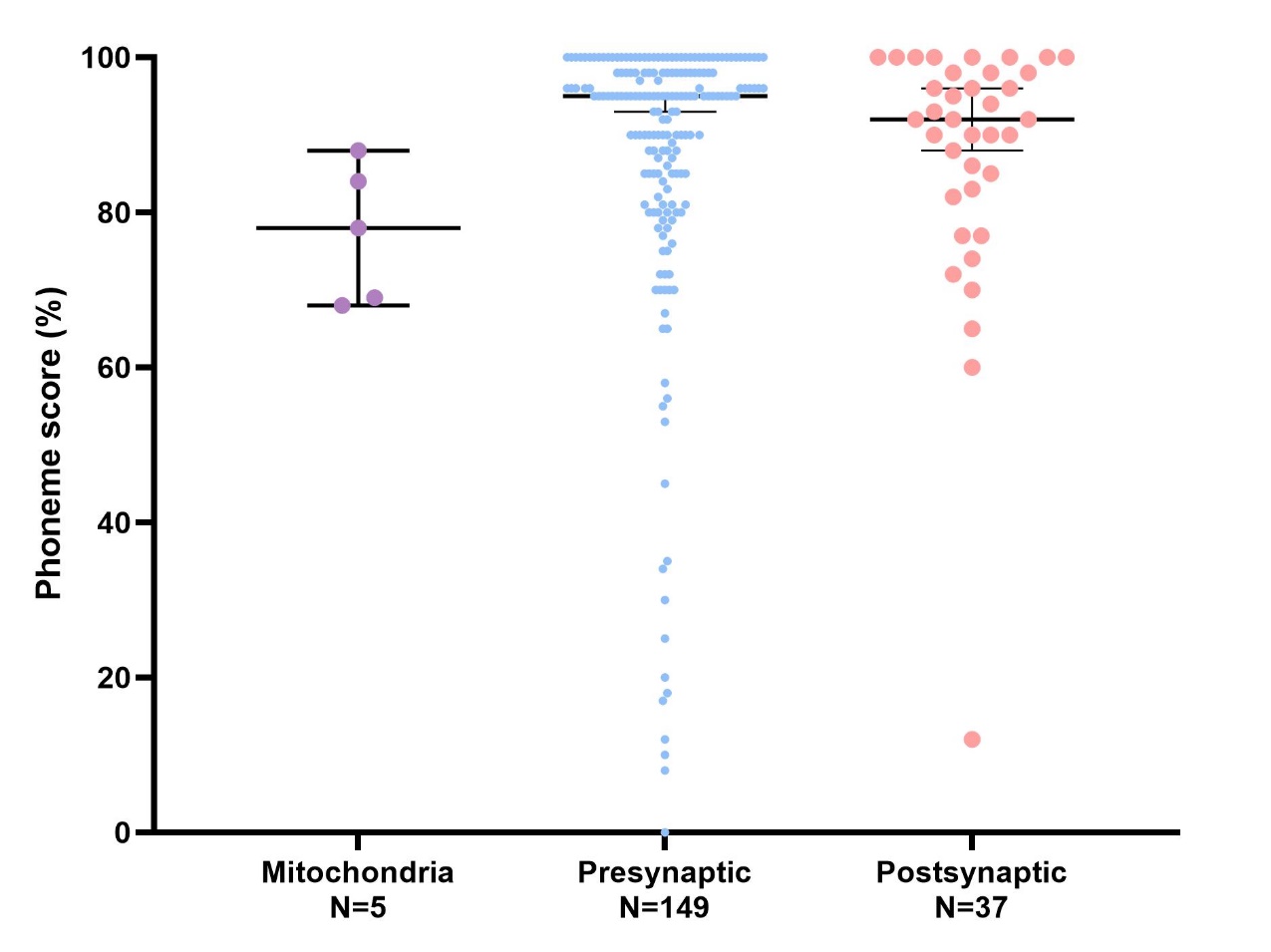


Scatterplots of the last measured phoneme scores at 65 dB SPL in quiet, broken down by subgroup. Each plot represents the median with interquartile ranges. There was no significant difference in the last measured phoneme score at 65 dB SPL between the pre-synaptic and post-synaptic group (p=0.594).
